# Hypoactivity of the Prefrontal Cortex During Go/No-Go Task in Patients With Generalized Anxiety Disorder

**DOI:** 10.1155/da/9040115

**Published:** 2025-07-28

**Authors:** Chien-An Chen, Po-Tsen Lin, Meng-Yu Hsu, Cheng-Yang Lee, I-Ming Chen, Yi-Ting Lin, Yu-Jui Huang, Pao-Huan Chen, Jia-Jin Chen, Gong-Hong Lin, Yi-Jing Huang

**Affiliations:** ^1^Department of Biomedical Engineering, National Cheng Kung University, Tainan, Taiwan; ^2^School of Occupational Therapy, National Taiwan University, Taipei, Taiwan; ^3^Lee Cheng-Yang Psychiatric Clinic, Taipei, Taiwan; ^4^Department of Psychiatry, National Taiwan University Hospital, Taipei, Taiwan; ^5^Department of Psychiatry, Taipei Medical University Hospital, Taipei, Taiwan; ^6^International Ph.D. Program in Gerontology and Long-Term Care, Taipei Medical University, Taipei, Taiwan; ^7^Department of Physical Medicine and Rehabilitation, National Taiwan University Hospital, Taipei, Taiwan

**Keywords:** fNIRS, generalized anxiety disorder, inhibition, prefrontal cortex

## Abstract

**Background and Objective:** Generalized anxiety disorder (GAD) is a prevalent mental health condition affecting cognitive functions like response inhibition. The neural mechanisms underlying the interplay between inhibitory ability and anxiety regulation in GAD remain unclear. This study aimed to investigate the prefrontal cortex (PFC) alterations when anxiety regulation shares neural resources with response inhibition in patients with GAD compared to healthy controls and to explore the relationship between anxiety and PFC activity.

**Methods:** The hemodynamic responses of bihemispheric PFC were measured in 19 GAD patients and 38 healthy controls using functional near-infrared spectroscopy (fNIRS) during the Go/No-Go task and were compared between the groups. The correlations between PFC activity and task performance and those between PFC activity and anxiety levels were analyzed.

**Results:** The GAD group exhibited lower hemoglobin concentration across the PFC during both baseline and task sessions, with significant hypoactivity in the bihemispheric dorsomedial PFC (DMPFC) at baseline (*p*=0.035–0.049), and more widespread hypoactivity during the task in the bihemispheric DMPFC (*p* < 0.001–0.033) and dorsolateral PFC (DLPFC; *p*=0.012–0.042), as well as the right ventromedial PFC (VMPFC; *p*=0.019–0.037). Higher baseline prefrontal activity was associated with poorer task accuracy (*r* = −0.576 to −0.417) and greater trait anxiety (*r* = 0.441–0.514). When transitioning to the task, better accuracy correlated with increased activation in the left DMPFC (*r* = 0.405–0.593), whereas higher anxiety levels were linked to reduced activation in the left DMPFC (*r* = −0.512) and right DMPFC (*r* = −0.435).

**Conclusion:** This study reveals that patients with GAD exhibit significant hypoactivity in the PFC during response inhibition, correlating with both task performance and anxiety levels. These findings emphasize the importance of targeting PFC dysfunction in the development of diagnostic tools and therapeutic interventions for GAD.

## 1. Introduction

Generalized anxiety disorder (GAD) is a prevalent mental health condition characterized by excessive, pervasive, and persistent worry about a wide range of events or activities. worry about various events or activities. It affects ~2.8% of adults annually and has a lifetime prevalence of 3.7% [1–3]. However, previous surveys revealed that more than 50% of the general population and 71% of primary care patients report symptoms consistent with GAD, despite lacking a formal diagnosis [4, 5]. Advancing our understanding of the neural mechanisms underlying GAD could lead to improvements in its diagnosis and treatment.

GAD profoundly impacts cognitive function, particularly response inhibition and other executive functions [6]. Some studies have found that patients with GAD tend to exhibit slower and less accurate responses on inhibition tasks compared to healthy controls [7]. This impairment may stem from the cognitive load imposed by excessive worry, which depletes attentional resources needed for effective task performance [8, 9]. Furthermore, impaired inhibition can contribute to the persistence of GAD symptoms by allowing excessive worry and threat-relevant information to dominate cognitive processes [10]. However, other studies have shown that patients with GAD exhibited better inhibition than healthy controls, and this increased inhibition is correlated with more severe GAD symptomatology [11–13]. The enhanced inhibition may result from the maladaptive use of inhibition control over threat processing as an avoidance strategy to minimize autonomic reactivity to phobic stimuli. In summary, the relationship between inhibitory control and GAD symptoms is complex and warrants further investigation to clarify these conflicting findings.

Anxiety regulation and cognitive function share neural capacity in the prefrontal cortex (PFC), which is markedly dysregulated in anxiety disorders [14, 15]. The ventromedial PFC (VMPFC) has been shown to be associated with emotional processing [16, 17], the dorsomedial PFC (DMPFC) appears to specialize in the appraisal of threats and distress tolerance [18, 19], and the dorsolateral PFC (DLPFC) is largely thought to regulate emotional processing in a top–down manner through its inhibitory connections to other brain regions, especially the amygdala [20]. In anxiety disorders, reduced VMPFC activation is linked to impaired fear extinction and maladaptive generalization, contributing to anticipatory anxiety [21]. The decreased VMPFC activation also impairs safety-threat differentiation and contributes to heightened threat perception [22]. The DMPFC integrates affective and cognitive components of emotional experience and is involved in encoding, consolidating, and retrieving emotionally salient memories [23]. Hyperactivation of the DMPFC and stronger positive coupling between the DMPFC and amygdala during fearful face processing have been reported in anxiety disorders, which may contribute to their heightened threat appraisal [24, 25]. Furthermore, studies have reported both hypoactivation and hyperactivation in the DLPFC of GAD patients during cognitive tasks. Some studies have shown hypoactivation of the DLPFC during emotion regulation tasks, suggesting that insufficient top-down control from the DLPFC may lead to excessive reactivity to negative stimuli [26]. Conversely, other research indicates GAD shows hyperactivation in the DLPFC when responding to angry expressions or threats relative to healthy controls [27, 28]. Still, another study has found no significant differences in frontoparietal activity during cognitive reappraisal between anxiety patients and healthy controls [29]. In summary, these neuroimaging findings highlight the complex and interconnected nature of anxiety regulation and cognitive function in GAD. Dysregulation in specific PFC subregions can contribute to attentional biases, impaired response inhibition, and difficulties in emotion regulation, which are hallmarks of GAD. However, the heterogeneous results suggest that the PFC's role in GAD is complex, varies depending on the specific task, and remains incompletely understood.

Functional near-infrared spectroscopy (fNIRS) during the Go/No-Go task offers a valuable approach for examining PFC alterations associated with the interplay between response inhibition and anxiety regulation in GAD. As a non-invasive and portable technique, fNIRS measures brain activity by detecting changes in blood oxygenation levels, allowing participants to remain in a more natural and relaxed environment, which is particularly beneficial for individuals with GAD [30]. Additionally, the Go/No-Go task is a well-known experimental paradigm of response inhibition, which is extensively utilized to elicit PFC activation [31, 32]. When performed under time pressure, the Go/No-Go task can intensify response inhibition difficulties and heighten anxiety. Thus, to fully investigate the PFC alterations when anxiety regulation shares neural resources with response inhibition, we aimed to investigate the PFC activity during the Go/No-Go task in patients with GAD compared to healthy controls and to explore the relationship between anxiety and PFC activity using fNIRS.

## 2. Materials and Methods

### 2.1. Participants

Patients with GAD were prospectively recruited from the department of psychiatry in two teaching hospitals and one psychiatry clinic in northern Taiwan between June 2023 and May 2024. Healthy controls were recruited from the local community close to the hospital using advertisements.

Patients were recruited if they met the following six inclusion criteria: (1) adults (aged 18 years or older) diagnosed with GAD according to the DSM-5 criteria by a psychiatrist; (2) native Mandarin Chinese speakers; (3) right-handed; (4) normal vision with or without correction; (5) normal hearing and verbal communication; and (6) regular follow-up visits for medication and/or psychotherapy during the study period. The inclusion criteria for healthy controls were as follows: (1) native Mandarin Chinese speakers; (2) right-handed; (3) normal vision with or without correction; and (4) normal hearing and verbal communication. Patients and healthy controls were excluded if any of the following criteria existed: (1) severe neurological disorders (e.g., stroke, brain tumor); (2) other major psychiatric disorders (e.g., other anxiety disorders, substance abuse, major depressive disorder, schizophrenia, bipolar disorder); and (3) severe or unstable medical conditions (e.g., acute gastrointestinal disease, cardiovascular disease, thyroid disease). The study was approved by the institutional review board of the aforementioned hospitals, and written informed consent was obtained from each participant.

### 2.2. Go/No-Go Task

The Go/No-Go task required participants to respond to a target stimulus (e.g., a blue circle) by pressing a single keyboard button and to withhold their response when presented with a nontarget stimulus (e.g., a green triangle). The target and nontarget stimuli were presented at a 3:1 ratio in the center of the screen.

**Figure 1:**
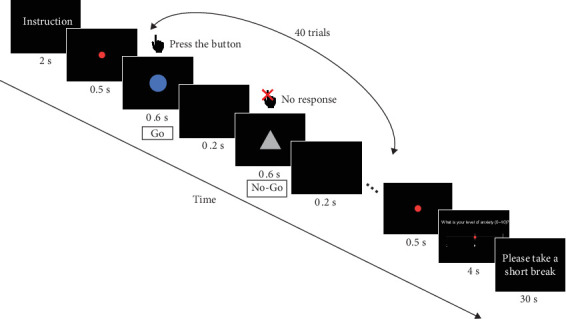
Example of the stimulus sequence in the Go/No-Go task.

Before the task began, participants were provided with task instructions, a demonstration, and a short practice session with fewer trials to familiarize themselves with the task. The main Go/No-Go task consisted of four blocks. Each block began with a 2-s display of the target stimulus instructions, followed by a 0.5-s red dot at the center of the screen, signaling the start of the task. During each trial, a stimulus was displayed for 0.6 s, with an inter-stimulus interval of 0.2 s, totaling 40 trials per block. At the end of each block, a 0.5-s red dot was displayed to signal the conclusion of the task. Afterward, participants rated their current level of anxiety using a Visual Analog Scale (VAS) for 4 s and took a 30-s rest before proceeding to the next block. The participants' overall response accuracy of Go and No-Go trials as well as reaction time on correct Go trials were recorded as task performance. The flow of the Go/No-Go task is shown in Figure 1.

### 2.3. fNIRS Measurement and Data Acquisition

**Figure 2:**
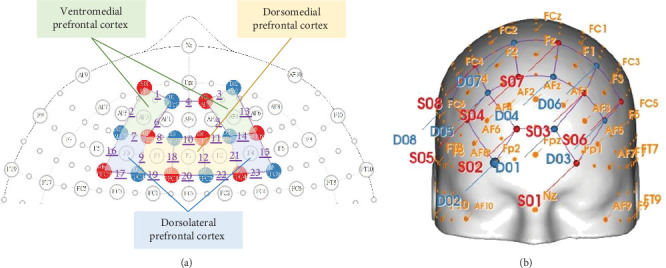
fNIRS montage. (a) Eight sources (red circles), eight detectors (blue circles), and 23 channels (purple numbers with underlines) on a standard 10-5 system. (b) Eight sources (red circles) and eight detectors (blue circles) onto a head model showing the coverage on the prefrontal cortex.

This study utilized a continuous-wave fNIRS device, NIRSport2 (NIRx Medizintechnik GmbH, Berlin, Germany), to collect hemodynamic responses in the bilateral PFC of participants during the Go/No-Go task. The fNIRS device operated with near-infrared light at two wavelengths (760 and 850 nm), sampling at a rate of 10.17 Hz. A total of 8 LED light sources and 8 detectors were positioned on the participants' scalps according to the international 10–5 standard electrode placement system, targeting the VMPFC, DLPFC), and DMPFC bilaterally (as illustrated in Figure 2). The source-detector separation was ~3 cm for each channel. The fNIRS signals were recorded using Aurora v2021.9 acquisition software. Signal quality was checked for all source-detector channels prior to measurement.

The fNIRS measurements were conducted in a quiet and dimly lit environment. Participants sat comfortably with a computer screen and a single-key keyboard placed in front of them. Throughout the fNIRS measurement, participants were instructed to remain seated, minimize head and body movement, and maintain their gaze at the central fixation cross on the computer screen to reduce motion artifacts and ensure accurate data collection.

The fNIRS measurement began with two baseline recordings, each lasting 120 s, separated by a 30-s rest period. During baseline recordings, participants were instructed to continuously press a single keyboard button. Two baseline measurements were performed to account for potential residual effects of brain activity induced by the preceding practice trials, which might influence the first baseline measurement. Following the baseline recordings, the Go/No-Go task was conducted to measure task-evoked brain activity.

### 2.4. fNIRS Signal Processing

The fNIRS signals were preprocessed using the open-source MATLAB application Homer3. Initially, the raw data were visually inspected for signal quality, artifacts, and channel connectivity, with any apparent sudden noise and poorly connected channels being removed. Then the raw light intensity data were converted into optical density. Next, a bandpass filter (0.001–0.1 Hz) was applied to remove physiological noise. The corrected optical density was subsequently transformed to the changes in concentration of oxygenated (ΔHbO) and deoxygenated hemoglobin using the modified Beer–Lambert Law [33]. In this study, the ΔHbO was selected as the indicator of cortical activity because it was found more sensitive than deoxygenated hemoglobin in reflecting task-induced changes in regional cerebral blood flow [34].

For each participant, the average ΔHbO was calculated for each channel during the baseline and task periods. ΔHbO from 5 s after the start to the end of the second baseline measurement was used for the baseline period, while ΔHbO from 5 s after the start to the end of each block of the Go/No-Go task was used for the task periods to account for delayed hemodynamic responses [35]. To address individual variability, ΔHbO values for each period were normalized using the average ΔHbO from the first 5 s of the respective period as a reference point for correction. The data from the four blocks of the Go/No-Go task were averaged to obtain an average response.

### 2.5. Clinical Anxiety Assessment

The Hamilton [36] Anxiety Rating Scale (HAM-A) is one of the most widely used clinician-administered psychological scales to assess the severity of anxiety symptoms. The scale includes 14 items reflecting anxiety symptoms of both psychic anxiety and somatic anxiety. Each item is rated on a scale from 0 to 4, and the total score ranges from 0 to 56. Higher scores indicate more severity of anxiety. HAM-A scores of 8–14 were classified as mild anxiety, 15–23 as moderate anxiety, and ≥24 as severe anxiety [37]. The reliability and the concurrent validity of the HAM-A were sufficient in anxiety disorders [38]. In this study, only patients with GAD were assessed with HAM-A.

The State-Trait Anxiety Inventory (STAI) is a commonly used self-report questionnaire of trait and state anxiety [39]. The STAI consists of 40 items, divided equally between the State Anxiety Scale (STAI-S) and the Trait Anxiety Scale (STAI-T). The STAI-S assesses the person's temporary emotional state, while the STAI-T measures the person's general tendency to experience anxiety across various situations. Each item is rated on a 4-point scale, with total scores ranging from 20 to 80 for each scale. Higher scores indicate greater levels of anxiety. The reliability and validity of the STAI to measure anxiety have been well documented [40].

The VAS was used for the participants to self-rate their level of anxiety at the moment of the Go/No-Go task. Participants were asked to mark their perceived anxiety level on a 10-cm horizontal line, where the far left represents the least anxiety and the far right represents the highest anxiety.

### 2.6. Statistical Analysis

Participant characteristics and the Go/No-Go task performance were analyzed using descriptive statistics and compared between patients with GAD and healthy controls using chi-square tests or independent *t*-tests.

The distributions of ΔHbO variables at baseline, task-state, and task-evoked conditions were examined for normality. All variables met acceptable criteria (absolute skewness <3 absolute kurtosis <10) [41], supporting the use of parametric tests. Cortical activation evoked by the Go/No-Go task was analyzed by comparing the average ΔHbO during the task period and that during baseline for every channel within each group by paired *t*-tests.

Cortical activity between patients with GAD and healthy controls was compared using independent *t*-tests. Welch's *t*-tests were applied when the assumption of homogeneity of variances was violated. The comparisons included between-group differences in baseline ΔHbO, task-state ΔHbO, and the task-evoked cortical activation (i.e., change in average ΔHbO from baseline to task) for each channel.

The relationship between cortical activity and anxiety, as well as between cortical activity and task performance, was analyzed within each group using Pearson's correlation coefficient. Specifically, correlations were assessed between the average ΔHbO during each session, the task-evoked cortical activation, and the HAM-A, STAI-S, STAI-T, VAS scores, the Go/No-Go task accuracy, and reaction time. A correlation was considered notable when |*r*| ≥0.40 [42].

The statistical analyses were conducted using SPSS (version 29; IBM Corp., Armonk, NY, USA). Significance was set at *p* < 0.05. For the fNIRS channel-wise analyses, the Benjamini–Hochberg false discovery rate (FDR) procedure was applied to adjust *p* values for the 23-channel multiple comparisons [43, 44].

## 3. Results

### 3.1. Participant Characteristics and Go/No-Go Task Performance

A total of 19 patients with GAD and 38 healthy controls participated in this study. Demographic and clinical characteristics of participants with GAD and healthy controls are summarized in Table 1. There were no significant differences between the groups in age, gender, educational level, marital status, or employment (*p*=0.206–0.784). On average, patients with GAD exhibited moderate anxiety, with a mean HAM-A score of 23.61. Significant differences were observed in the VAS, STAI-S, and STAI-T scores, with patients reporting higher level of anxiety at the moment of the Go/No-Go task (*p*=0.048) as well as state and trait anxiety levels (*p* < 0.001). Additionally, patients with GAD had an average accuracy of 80.85% and a mean reaction time of 0.41 s during the Go/No-Go task. These performance metrics were comparable to those of healthy controls, with no significant differences in accuracy (*p*=0.251) or reaction time (*p*=0.130).

### 3.2. Cortical Activation Evoked by Go/No-Go Task Within Each Group

**Figure 3:**
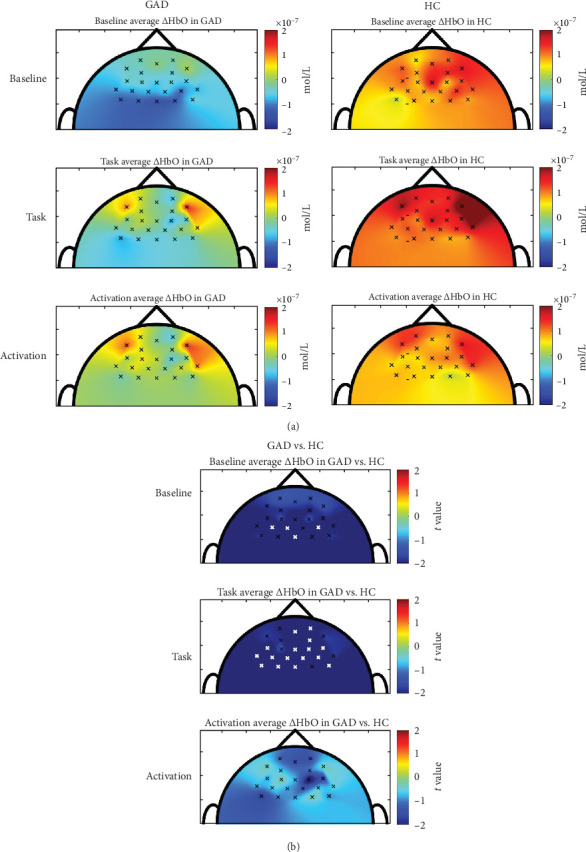
(a) Topographic maps of average ΔHbO during baseline session (first row), average ΔHbO during the Go/No-Go task session (second row), and task-evoked cortical activation (third row) in the GAD group (first column) and healthy controls (second column). (b) t-map for between-group differences in ΔHbO during baseline session (first row), average ΔHbO during the Go/No-Go task session (second row), and task-evoked cortical activation (third row). White crosses indicate significant differences between groups. GAD, generalized anxiety disorder; HC, healthy controls.

No channel exhibited a significant change in average ΔHbO between baseline and task sessions in either the GAD group (FDR-adjusted *p*=0.575–1.000) or the healthy controls (FDR-adjusted *p*=0.936–1.000).  Figure 3 a presents topographic maps of baseline ΔHbO task-state ΔHbO, and the task-evoked cortical activation. Overall, the GAD group showed a trend of increased ΔHbO in the left VMPFC, right VMPFC and right DLPFC and decreased ΔHbO in the left DLPFC and right DMPFC from baseline to task, whereas the healthy controls exhibited increased ΔHbO across the bihemispheric PFC, particularly in the left VMPFC, right VMPFC, right DLPFC, and right DMPFC.

### 3.3. Comparison of Cortical Activity Between GAD and Healthy Controls

Figure 3 b displays the *t*-map for between-group differences in average ΔHbO. Overall, the GAD group exhibited lower ΔHbO across the bihemispheric PFC during both the baseline and the Go/No-Go task sessions compared to healthy controls. Specifically, significant hypoactivity at baseline was observed in the bihemispheric DMPFC (FDR-adjusted *p*=0.035–0.049). During the Go/No-Go task, more widespread and significant hypoactivity was found in the bihemispheric DMPFC (FDR-adjusted *p* < 0.001–0.033), bihemispheric DLPFC (FDR-adjusted *p*=0.012–0.042), and right VMPFC (FDR-adjusted *p*=0.019–0.037). However, the task-evoked cortical activation did not differ significantly between groups (FDR-adjusted *p*=0.299–0.983), although the GAD group showed a trend of reduced activation across the bihemispheric PFC compared to healthy controls.

### 3.4. Correlation of Task Performance, Anxiety, and Cortical Activity Within Each Group

**Figure 4:**
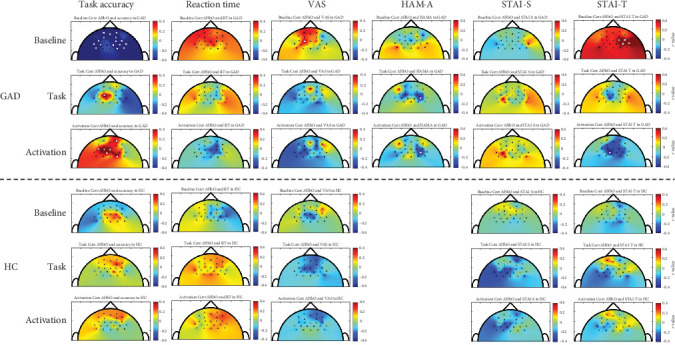
Correlation map between task performance, anxiety measures, and ΔHbO during the baseline session, task session, and cortical activation in the GAD group (top three columns) and healthy controls (bottom three columns). GAD, generalized anxiety disorder; HAM-A, Hamilton anxiety rating scale; HC, healthy controls; STAI-S, state-trait anxiety inventory-state anxiety scale; STAI-T, state-trait anxiety inventory-trait anxiety scale; VAS, visual analog scale.

Figure 4 illustrates the correlations between task performance and ΔHbO, as well as those between anxiety measures and ΔHbO in each group. In the GAD group, notable negative correlations were observed between the Go/No-Go task accuracy and baseline ΔHbO in multiple channels within the bihemispheric DLPFC (*r* = −0.525 to −0.417), bihemispheric VMPFC (*r* = −0.576 to −0.417), and right DMPFC (*r* = −0.525 to −0.432). Conversely, the Go/No-Go task accuracy was positively correlated with task-state ΔHbO in the left DMPFC (*r* = 0.580), as well as with task-evoked cortical activation in the left DMPFC (*r* = 0.405–0.593), right VMPFC (*r* = 0.402), and right DMPFC (*r* = 0.451). In terms of anxiety measures, VAS scores showed a positive correlation with baseline ΔHbO in left DMPFC (*r* = 0.430) and STAI-T scores were positively correlated with baseline ΔHbO in several channels of the right DLPFC (*r* = 0.441–0.514) and right DMPFC (*r* = 0.505). A notable positive correlation was observed between the HAM-A scores and both task-state ΔHbO (*r* = −0.451) and task-evoked cortical activation in right DMPFC (*r* = −0.435). In addition, the STAI-T scores were negatively correlated with task-evoked cortical activation in the left DMPFC (*r* = −0.512). In healthy controls, no notable correlations were identified between task performance or anxiety measures and any of the ΔHbO measures (baseline, task-state, or task-evoked cortical activation).

## 4. Discussion

This study is pioneering in exploring the potential neural mechanisms underlying the interplay between inhibitory ability and anxiety regulation in patients with GAD using fNIRS. The results revealed that, compared to healthy controls, the GAD group exhibited consistently lower ΔHbO across the bihemispheric PFC during both baseline and the Go/No-Go task sessions. Significant hypoactivity was particularly evident at baseline in the bihemispheric DMPFC and became more widespread during the task, extending to the bihemispheric DLPFC, bihemispheric DMPFC, and right VMPFC. Although task-evoked cortical activation did not differ significantly between groups, a general trend of reduced activation in the GAD group was observed. Interestingly, patients with GAD performed the Go/No-Go task with comparable accuracy and reaction time to healthy controls, despite reporting significantly higher levels of state, trait, and momentary anxiety. This dissociation between neural hypoactivity and preserved task performance may point to compensatory neural strategies that enable individuals with GAD to maintain behavioral performance and regulate heightened anxiety despite reduced prefrontal activation.

The significant differences in ΔHbO values levels between the GAD group and healthy controls during both the baseline and the Go/No-Go task sessions indicate distinct neural processing patterns associated with anxiety. At baseline, the lower ΔHbO values observed in the GAD group suggest inherent hypoactivity in the bihemispheric DMPFC, potentially reflecting chronic under-engagement of regions involved in threat appraisal and emotionally salient memories, even in the absence of explicit task demands. During the Go/No-Go task, which requires response inhibition, this hypoactivity became more widespread, extending to the bihemispheric DLPFC, bihemispheric DMPFC, and right VMPFC, which are regions critical for executive function and emotional regulation. This persistence and expansion of hypoactivity across both resting and task-engaged states underscores a potential underlying dysfunction in the neural circuitry of individuals with GAD, which may impair their ability to engage these regions effectively, regardless of the task demands. Reduced activity in these PFC regions may indicate a dysfunctional neural circuitry that impairs the ability to regulate anxiety and inhibit response properly.

However, when examining task-evoked cortical activation, no significant differences were found between the groups. This suggests that while the overall PFC activity was reduced in GAD group during specific states, the relative change in task-evoked activation is comparable to that of healthy controls, primarily showing increased ΔHbO in the bihemispheric VMPFC and right DLPFC. Our findings align with a previous study [45] that also found no significant differences in PFC activation elicited by the Go/No-Go task between the GAD and healthy controls, despite comparable task accuracy. The preserved task-evoked response in GAD, despite reduced baseline activity, may reflect compensatory neural mechanisms that support performance under cognitive demands. However, such compensation may come at a cost, as individuals with GAD must also regulate elevated anxiety, placing additional strain on neural systems involved in both cognitive control and emotional regulation.

A closer examination of task-evoked activation patterns revealed group-specific differences in the right DMPFC and left DLPFC. In healthy controls, activation tended to increase in both regions from baseline to task, whereas in the GAD group, these regions showed a trend of deactivation. This divergence suggests that while general activation patterns may appear similar at the group level, GAD patients may exhibit altered or insufficient engagement of specific prefrontal regions critical for inhibitory control. The reduced engagement of the left DLPFC and deactivation of the right DMPFC may reflect anxiety-related disruptions in the dynamic recruitment of executive networks. These findings align with previous studies showing lower left DLPFC activation in GAD during working memory tasks with emotional distractors [46] and greater right DLPFC activation in GAD during high-load tasks, suggesting a compensatory shift in lateralization [47]. Additionally, a meta-analysis of fMRI studies found reduced bilateral DMPFC activation during cognitive reappraisal in individuals with anxiety disorders compared to healthy controls [48]. Collectively, these findings reinforce the atypical recruitment and even deactivation of key prefrontal regions, particularly the right DMPFC and left DLPFC, could contribute to compromised cognitive and emotional processing in GAD.

Our correlational results also revealed distinct neural mechanisms underlying task performance and anxiety regulation in individuals with GAD. Specifically, higher baseline ΔHbO in bihemispheric DLPFC, bihemispheric VMPFC, and right DMPFC was negatively correlated with the Go/No-Go task accuracy, suggesting that elevated resting-state prefrontal activity may reflect inefficient or maladaptive preparatory processes that interfere with cognitive control during subsequent task engagement. In terms of anxiety regulation, trait anxiety was positively correlated with baseline ΔHbO in the right DLPFC and right DMPFC, while higher VAS scores were associated with increased baseline ΔHbO in the left DMPFC. These findings support the notion that prefrontal hyperactivity at rest, particularly in the DMPFC and DLPFC, may represent a neural signature of chronic hyperarousal and subjective anxiety in GAD. These associations imply that baseline hyperactivity in specific prefrontal regions may reflect a neural signature of persistent anxiety in GAD. Taken together, these baseline findings indicate that pretask hyperactivity in specific prefrontal areas may both reflect and contribute to inefficient cognitive functioning and persistent anxiety in GAD.

During the Go/No-Go task, higher task accuracy in the GAD group was positively associated with task-state ΔHbO in the left DMPFC and with task-evoked cortical activation in the left DMPFC, right VMPFC, and right DMPFC. These results highlight the potential compensatory role of the DMPFC and VMPFC in supporting inhibitory control during cognitive demand. However, trait anxiety was negatively associated with task-evoked activation in the left DMPFC, and higher HAM-A scores were also associated with reduced task-evoked activation in the right DMPFC and lower task-state ΔHbO. These associations suggest that elevated anxiety may hinder the effective engagement of prefrontal regions, thereby compromising their compensatory function during task execution. This pattern suggests a potential dual role of the DMPFC, as a region that may support cognitive inhibition and one that may be vulnerable to anxiety-related interference, highlighting its possible relevance for therapeutic considerations in GAD.

This study has several limitations that should be considered. Firstly, the sample size was relatively small, which may limit the generalizability of the findings. Secondly, fNIRS has methodological constraints, as it indirectly measures cortical activity through blood oxygenation changes, which may not precisely reflect neural activity. Its spatial resolution is also lower than fMRI, potentially missing finer brain activity details. Thirdly, although most GAD participants were under pharmacological treatment, the study did not systematically record details regarding medication type, dosage, or treatment duration. These unmeasured pharmacological factors could have influenced prefrontal activation and task performance. In addition, the study design did not account for potential confounding variables such as individual differences in cognitive strategies, which might also have influenced the results. Lastly, the cross-sectional nature of the study prevents us from drawing causal conclusions about the observed neural patterns and their role in GAD. These limitations highlight the need for further research to validate and extend the findings of this study.

## 5. Conclusions

This study explored the neural mechanisms underlying the interplay between inhibitory ability and anxiety regulation in patients with GAD using fNIRS. The findings revealed that individuals with GAD exhibited hypoactivity across the bihemispheric PFC during both baseline and the Go/No-Go task sessions, indicating distinct neural processing patterns associated with pathological anxiety. Despite this hypoactivity, GAD patients maintained task performance levels, suggesting compensatory mechanisms. The findings highlight the potential dysfunction in the prefrontal regions, which are crucial for executive functions and emotional regulation. Additionally, higher anxiety levels in GAD patients were associated with increased baseline activity in the right DLPFC and DMPFC but reduced task-evoked activation in the bihemispheric DMPFC, suggesting that elevated anxiety may disrupt the flexible engagement of prefrontal regions essential for inhibitory control. Understanding these divergent neural pathways could inform personalized therapeutic strategies aimed at enhancing cognitive function and emotional regulation in GAD patients.

## Figures and Tables

**Table 1:** Demographic and clinical characteristics of patients with generalized anxiety disorder and healthy controls.

Characteristics	GAD (*n* = 19)	HC (*n* = 38)	GAD vs. HC (*p*-Value)
Age (years)	52.68 ± 14.79	59.03 ± 22.24	0.206
Gender (female/male)	14/5	25/13	0.546
Education level (≤high school/≥college)	6/12	11/27	0.739
Marriage (single or divorced/married, separated, or widowed)	6/12	13/22	0.784
Employment (full-time or part-time or volunteer/unemployed or homemaker)	9/9	16/19	0.767
Therapy (medication/psychotherapy/both)	16/1/2	—	—
Go/No-Go task accuracy (%)	80.85 ± 6.46	78.35 ± 9.67	0.251
Go/No-Go task reaction time (s)	0.41 ± 0.03	0.39 ± 0.03	0.130
VAS score	3.30 ± 1.76	2.22 ± 1.97	0.048
HAM-A score	23.61 ± 8.35	—	—
STAI-S score	46.11 ± 9.48	32.13 ± 8.55	<0.001
STAI-T score	58.79 ± 8.99	37.87 ± 10.95	<0.001

*Note:* Values are means ± standard deviations.

Abbreviations: GAD, generalized anxiety disorder; HAM-A, Hamilton Anxiety Rating Scale; HC, healthy controls; STAI-S, state-trait anxiety inventory-state anxiety scale; STAI-T, state-trait anxiety inventory-trait anxiety scale; VAS, visual analog scale.

## Data Availability

The data that support the findings of this study are available upon request from the corresponding author. The data are not publicly available due to privacy or ethical restrictions.
